# Supplementary material for: How does COVID-19 vaccination affect long-COVID symptoms?
Source: PLoS One. 2024 Feb 7;19(2):e0296680. doi: 10.1371/journal.pone.0296680 (PMC10849259; doi:10.1371/journal.pone.0296680)
Supplement: S1 Appendix — (DOCX) [file pone.0296680.s002.docx]

**Appendix 1.** Long long-COVID and vaccine study, Fars, Iran.

Name: Sex: male /female Phone number:

Have you experienced another episode of COVID-19 after your initial infection (any infection after we called you last year)? Yes / No

Have you received a COVID-19 vaccine? No / 1 dose / 2 doses / 3 doses

- **Have experienced any symptoms or complaints or problems during the past week (any symptoms or complaints or problems that you did not have before your COVID-19, but have had ever since after your illness and specifically during the past seven days)?**

1. Muscle weakness Yes /No

2. Muscle pain Yes /No

3. Joint pain Yes /No

4. Fatigue Yes /No

5. Sleep difficulty Yes /No

6. Shortness of breath Yes /No

7. Chest pain Yes /No

8. Palpitation Yes /No

9. Cough Yes /No

10. Excess sputum Yes /No

11. Decreased sense of smell Yes /No

12. Decreased sense of taste Yes /No

13. Sore throat Yes /No

14. Headache Yes /No

15. Dizziness Yes /No

16. Concentration difficulty Yes /No

17. Excess sweating Yes /No

18. Exercise difficulty Yes /No

19. Walking difficulty Yes /No

20. Diarrhea Yes /No

21. Abdominal pain/stomach ache Yes /No

22. Loss of appetite Yes /No

23. Weight loss Yes /No

Amount…...Kg

24. Weight gain Yes /No

Amount……Kg

25. Please mention other symptoms or complaints ….
