# Supplementary material for: How does COVID-19 vaccination affect long-COVID symptoms?
Source: PLoS One. 2024 Feb 7;19(2):e0296680. doi: 10.1371/journal.pone.0296680 (PMC10849259; doi:10.1371/journal.pone.0296680)
Supplement: Attachment — Submitted filename: response letter.docx [file pone.0296680.s004.docx]

Dear Editor in Chief,

We are so thankful to the reviewers and editorial team for their insightful comments that helped us to improve the current version.

Associated Editor Comments

1-Please ensure that your manuscript meets PLOS ONE's style requirements, including those for file naming.

Thanks for pointing it out. We checked our manuscript and prepared it to meet the journal's guidelines.

2. We noticed you have some minor occurrences of overlapping text with the following previous publication(s), which needs to be addressed:
- <https://www.science.org/content/article/rare-cases-coronavirus-vaccines-may-cause-long-Covid-symptoms>
In your revision ensure you cite all your sources (including your own works), and quote or rephrase any duplicated text outside the methods section. A further consideration is dependent on these concerns being addressed.

The mentioned website considered seventeen citations of our manuscript. We have reviewed our manuscript again.

3. In the ethics statement in the Methods, you specified that verbal consent was obtained. Please provide additional details regarding how this consent was documented and witnessed, and state whether this was approved by the IRB.

Our manuscript was approved by the Ethical Committee of Shiraz University of Medical Sciences, Shiraz, Iran (IR.SUMS.Rec.1399.022 & m/h/19/91/8225). As the data was obtained by phone, we asked our patients for consent. If they agreed, we continued to interview.

4. Please include a complete copy of PLOS’ questionnaire on inclusivity in global research in your revised manuscript. Our policy for research in this area aims to improve transparency in the reporting of research performed outside of the researchers’ own country or community. The policy applies to researchers who have traveled to a different country to conduct research, research with Indigenous populations or their lands, and research on cultural artifacts. The questionnaire can also be requested at the journal’s discretion for any other submissions, even if these conditions are not met.  Please find more information on the policy and a link to download a blank copy of the questionnaire here: <https://journals.plos.org/plosone/s/best-practices-in-research-reporting>. Please upload a completed version of your questionnaire as Supporting Information when you resubmit your manuscript.

Thanks for suggesting this issue, we are uploading a completed version of the questionnaire.

5. We note that you have stated that you will provide repository information for your data at acceptance. Should your manuscript be accepted for publication, we will hold it until you provide the relevant accession numbers or DOIs necessary to access your data. If you wish to make changes to your Data Availability statement, please describe these changes in your cover letter and we will update your Data Availability statement to reflect the information you provide.

OK.

6. Please include captions for your Supporting Information files at the end of your manuscript, and update any in-text citations to match accordingly. Please see our Supporting Information guidelines for more information: <http://journals.plos.org/plosone/s/supporting-information>.

Supporting information files are added at the end of our manuscript.

Reviewers comment

1- The Abstract must report the aim of the study, the basic information on the sample (time span, countries analyzed), the empirical methodology used, the main findings, and the relevant policy implications.

Thank you for your comment. The aim of the study has been written in simple words in order to clarify the concept better.

2-Introduction and Literature Review should be split into two different sections. The Introduction should highlight the relevance of the topic, the novelty of the results, the importance of policy implications, the sample’s choice, the methodology’s appropriateness, the data used, the contribution to the literature, and the limitations of the study.

As suggested by the reviewer. In the revised manuscript, both sections are covered in the introduction part.

3-The literature review is partial and incomplete, and some recent and relevant contributions should be cited and discussed: i.e., 10.1017/S0950268822001418.

New recent and relevant contributions are added to our article.

4-The theoretical framework should be discussed more in detail.

Revised.

5-The choice of methodology needs to be clearly stated and motivated.

Revised.

6-The estimated model must be justified in light of the literature on this specific topic.

There was no model.

7-Data should be defined more clearly. A link to the data source must be reported.
see the data availability statement.

8-Descriptive statistics are absent.

As it is mentioned, In total, 1988 people were included in the current study [1041 male (52.4%) and 947 female (47.6%) participants]. The median age of the participants was 52 years (minimum: 18, maximum: 97, and interquartile range: 23 years). Of the studied participants, 1236 people (62.2%) reported having long COVID in phase 1 of the study; these were selected for the next steps of the analyses.

9-Diagnostic tests are absent.

Thank you for your comment, we have added the PCR test as a diagnostic test in the method part.

10-Robustness checks are absent.

Not clear.

11-The results should be discussed more in detail.

Revised.

12-Comparisons with previous studies are absent.

We added some more comparisons to clarify our concept better. Some recent studies are added in the discussion and introduction parts.

13-Conclusions are too short.

We have changed the conclusion and discussed that in more detail.

14-Policy implications are weak.

Revised.

15-Further research should be indicated.

revised.

16-Limitations of the study are not provided.

Limitations of our study were added.

17-Proofreading by a native speaker is required.

All spelling and grammatical errors have been corrected.

18-The editing does not follow the journal’s guidelines.

We have checked our manuscript and revised our manuscript to follow the journal’s guidelines.

19-Some typos must be fixed.

We have fixed the typos.

20-How does the paper enrich the knowledge of the scientific community?

See the conclusion.
