# Supplementary material for: How does COVID-19 vaccination affect long-COVID symptoms?
Source: PLoS One. 2024 Feb 7;19(2):e0296680. doi: 10.1371/journal.pone.0296680 (PMC10849259; doi:10.1371/journal.pone.0296680)
Supplement: Attachment — Submitted filename: response letter 2.docx [file pone.0296680.s005.docx]

Dear Editor in Chief,

We are so thankful to the reviewers for their insightful comments that helped us to improve the current version.

Reviewer #3:

1. Results section was well described but it would be clearer if the authors show the results especially specific symptoms (listed in the appendix 1) in the table to let the audience sees the differences of long-covid symptoms among participants groups. e.g. vaccinated vs unvaccinated, reinfection.

**Response.** A table is included as suggested.

2. Logistic regression could also be applied to study the factors that could contribute to the long-long COVID symptoms although no control in the study but at least between persistent and non-persistent long-long COVID.

**Response.** While this is a valuable comment, it is not related to the question of our work (the longevity of symptoms associated with long COVID in adults in association with their COVID vaccination status). Besides, the groups were very imbalanced (51 vs 1185) for detailed statistical analyses with other tests.
