# Supplementary material for: How does COVID-19 vaccination affect long-COVID symptoms?
Source: PLoS One. 2024 Feb 7;19(2):e0296680. doi: 10.1371/journal.pone.0296680 (PMC10849259; doi:10.1371/journal.pone.0296680)
Supplement: Attachment — Submitted filename: response letter 3.docx [file pone.0296680.s006.docx]

**Dear Editor in Chief,**

We are thankful to the reviewers for their insightful comments that helped us to improve the current version.

Odds ratios (ORs) and 95% confidence intervals (CIs) were calculated by logistic regression test.

When binary logistic regression test was applied (vaccination status and sex as covariates and experiencing long long-COVID as the dependent variable), sex was not associated with experiencing long long-COVID (p = 0.454) while receiving any dose of COVID vaccine was significantly associated with long long-COVID (p = 0.032; OR = 1.96; 95% CI: 1.06-3.63).

Table 1 is revised with percentages included.
